# Associations between vitamin C intake and serum uric acid in US adults: Findings from National Health and Nutrition Examination Survey 2011–2016

**DOI:** 10.1371/journal.pone.0287352

**Published:** 2023-10-13

**Authors:** Yanting Yang, Sijie Zheng, Yunfu Feng

**Affiliations:** 1 Department of Gastroenterology, The Third People’s Hospital of Kunshan, Kunshan, China; 2 Endoscopy Center, The First People’s Hospital of Kunshan, Kunshan, China; Kerman University of Medical Sciences Physiology Research Center, ISLAMIC REPUBLIC OF IRAN

## Abstract

**Backgrounds:**

Dietary factors has been found to influence serum uric acid (SUA) levels. We further explored the associations between dietary and supplemental vitamin C intake and SUA in a large population-based study.

**Methods:**

The cross-sectional study included 6308 participants (3146 males and 3162 females) aged ≥20 years from the National Health and Nutrition Examination Survey (NHANES) 2011–2016 in the United States. The dietary vitamin C was log-transformed for statistical analysis. Hyperuricemia was defined as SUA concentrations >420 umol/L in males or >360 umol/L in females. The associations of dietary vitamin C and supplemental vitamin C with SUA levels and hyperuricemia risk were evaluated using weighted linear regression models and weighted multivariate logistic regression models, and a subgroup analysis stratified by gender was also conducted.

**Results:**

In this large-scale database study, there was a negative association between dietary vitamin C (log transformed) and SUA levels in US adults (β = −7.27, 95% CI: −11.58, −2.97). The inverse relationship existed among males but not females (P for interaction = 0.02). There was inverse correlation between dietary vitamin C (log transformed) and hyperuricemia risk (OR = 0.68, 95% CI: 0.57, 0.81), especially in males compared to females determined through an interaction test (P = 0.04). There were no associations between supplemental vitamin C and SUA levels (β = 1.00 (95% CI: −4.44, 6.44) or hyperuricemia risk (OR = 0.98 (95% CI: 0.78, 1.24). High-dosage supplemental vitamin C (>300 mg) and hyperuricemia risk were not associated (OR = 1.04, 95% CI: 0.69, 1.56).

**Conclusions:**

This study demonstrated that there were negative associations between dietary vitamin C and SUA levels and hyperuricemia risk among US adults. The inverse correlations between dietary vitamin C and hyperuricemia risk were more significant in males compared to females. There were no associations between supplemental vitamin C and SUA levels or hyperuricemia risk.

## Introduction

Serum uric acid (SUA), a natural antioxidant that helps humans resist antioxidant stress, is produced by purine metabolism [1, 2]. The links between elevated SUA levels or hyperuricemia are closely correlated with many diseases, such as gout [3, 4], hypertension [5, 6], diabetes [7, 8], cardiovascular diseases [9, 10] and chronic kidney disease etc. [11, 12]. The cutoff value at which uricemia becomes abnormal is still disputed [13]. The prevalence of hyperuricemia differs significantly across the world. In a representative Australian population, hyperuricemia prevalence varied between 10.5% and 16.6% [14], and there was a prevalence of 20.0%-20.2% among US adults in 2015–2016 [15]. In the Chinese population, there was a prevalence of 15.1% among males and 5.8% among females in adults between 18 and 59 years of age [16]. Regarding the high prevalence of hyperuricemia globally, lifestyle has an impact on the developmental process of hyperuricemia [17].

Studies had found that lifestyle factors, including diet, influenced SUA levels [18]. Vitamin C (VC) may be a dietary factor leading to lower SUA [19]. Human who cannot synthesize vitamin C depends on dietary sources to fulfill nutrient needs [20]. The associations of dietary VC intake and supplemental VC intake with SUA levels were suggested by several reports. A Korean study found total VC intake correlated negatively with SUA levels, but not dietary VC intake [21]. In the United States, the consumption of VC was negatively related with SUA concentrations in men with BMI < 30 kg/m^2^ and without hypertension [22]. A meta-analysis showed supplemental vitamin C reduced SUA levels [23]. However, a pilot randomized controlled trial found 500 mg/day vitamin C for 8 weeks had no clinically significant urate-lowering effects in patients [24].

The prevention and treatment of high SUA levels, which are the foundation of a series of diseases, have been among the major public health issues [25, 26]. Thanks to the new hypouricemic agents, hyperuricemia now can be controlled more easily [27]. Nevertheless, the significance of nonpharmacological treatment such as changing dietary habits is also a key factor [17]. We conducted a cross-sectional study on this topic to investigate the associations of dietary vitamin C and supplemental vitamin C with SUA levels and hyperuricemia risk in US adults aged ≥20 years as part of large population-based study from NHANES.

## Methods

### Study population

Information on study from the National Health and Nutrition Examination Survey (NHANES) assessed the associations of vitamin C intake with SUA levels and hyperuricemia risk. NHANES is a national survey of United States performed by the Centers for Disease Control and Prevention (CDC) to evaluate the status of health and nutrition of all Americans. Every two years, the data are released for research purposes. The design details can be searched at www.cdc.gov/nchs/nhanes/. NHANES data collected through in-person interviews and physical examinations performed in mobile examination centers are collected from demographic data, dietary data, examination data, laboratory data, and questionnaire data. The NHANES protocol was approved by the Ethics Review Board of the National Center for Health Statistics Research; all participants signed an informed consent document.

This research used NHANES data from 2011 to 2016, spanning three biennial circles of data collection (N = 29,902). The exclusion criteria of this study included participants under 20 years old (N = 12,854); pregnant women (N = 977); those with cancer, malignancy, or kidney disease (N = 1417); and those taking medications influenced SUA concentrations, such as diuretics (hydrochlorothiazide or furosemide) or hypouricemic agents (allopurinol or febuxostat) (N = 1060) (29). Individuals with missing essential data such as vitamin C intake (N = 3399) and serum uric acid (N = 3887) were also excluded. After excluding these subjects, 6308 participants (3146 males and 3162 females) aged ≥20 years old (from 20 to 80 years old) enrolled in the final analysis.

### Vitamin C intake measurement and SUA measurement

Information on the dietary vitamin C intake from the participants of NHANES was obtained by a 24-h dietary interview. Based on the dietary intake data, an estimation of food and beverage consumption during 24 hours before the interview was conducted. Food components, nutrients and energy could thus be calculated. Supplemental vitamin C data on vitamins, minerals, herbs, and other dietary supplements gathered by a 24-h dietary supplements interview. The information on dietary and supplemental VC intake was collected personally in the mobile examination center (MEC) in the first 24-h dietary recall. The vitamin C was composed of dietary VC and supplemental VC. The concentration of SUA was measured in NHANES Laboratory all three cycles. Hyperuricemia was defined as SUA concentrations >420 umol/L in males or >360 umol/L in females [21, 28].

### Variables

The continuity variables included age, body mass index (BMI), serum creatinine, serum triglycerides, serum cholesterol, dietary energy, dietary protein, dietary sugars, dietary fiber, dietary vitamin B6, dietary folate, and physical activity [29]. Physical activity was determined by multiplying the weekly amount of time spent in each activity by the metabolic equivalent of the task (MET).

The categorical variables included gender, race/ethnicity, educational level, marital status, smoking habits, alcohol consumption, hypertension, and diabetes. Race was self-reported as non-Hispanic white, non-Hispanic black, Mexican American, and other races including other Hispanic, non-Hispanic Asian, Multi-Racial et al. Education levels was classified into four grades: less than high school, high school graduate or equivalent, college or AA degree and college graduate or above. Marital status was grouped into married or living with a partner and living alone. Smoking habits were divided as never, current, or former smoker. Alcohol consumption based on at least 12 alcohol drinks in the last year was classified as non-drinkers or drinkers. Hypertension and diabetes were based on self-reported.

### Statistical analysis

The continuity variables were reported as the means ± standard deviation (SD), the differences were calculated by weighted linear regression models. Categorical variables were reported as counts and weighted percentages, and different characteristics were calculated using a weighted chi-squared test. Because dietary VC were not normally distributed, we used log transformation for statistical analysis. All analysis was performed with NHANES sample weights. Weighted multivariate linear regression models were performed to assess the associations between VC intake and SUA concentrations. Weighted multivariate logistic regression models were used to examine the associations between VC intake and hyperuricemia risk. Interaction tests were also calculated to examine the effect. Additional covariates were treated as potential effect modifiers. The optimal regression model was selected to analyses the smooth fitting curve. A two-sided p < 0.05 was indicated as a statistically significant difference. All statistical data were analyzed using R (http://www.R-project.org, The R Foundation, Boston, MA, USA) and EmpowerStats software (http://www.empowerstats.com, X&Y Solutions, Inc., Boston, MA, USA).

## Results

As shown in Table 1, this study included 6308 adults, which consisted of 3146 males and 3162 females. The mean dietary VC intake was 81.66 ± 91.27mg, 83.45 ± 94.96 mg for males and 79.90 ± 87.46 mg for females. The mean serum uric acid was 315.42 ± 80.62 umol/L, 316.50 ± 80.77 umol/L for males and 314.35 ± 80.46 umol/L for females. There was no significant difference between males and females in dietary vitamin C, supplemental vitamin C, serum uric acid, race, BMI, creatinine, triglycerides, cholesterol, dietary energy, dietary protein, dietary sugars, dietary vitamin B6, dietary folate, physical activity, hypertension and diabetes. Significant differences were shown in terms of hyperuricemia, age, education, dietary fiber, alcohol consumption and smoking habits.

**Table 1:** Characteristics of the study participants.

	Total (N = 6308)	Males (N = 3146)	Females (N = 3162)	*p* Value
Dietary vitamin C (mg)	81.66 ± 91.27	83.45 ± 94.96	79.90 ± 87.46	0.13
Supplemental vitamin C (mg)	70.56 ± 277.45	76.80 ± 327.06	64.44 ± 217.94	0.08
Serum uric acid (umol/L)	315.42 ± 80.62	316.50 ± 80.77	314.35 ± 80.46	0.30
Hyperuricemia (%)	18.23	10.21	26.09	<0.01
Serum uric acid >420 (umol/L)	10.13	10.21	10.07	0.85
Age (years)	45.49 ± 16.61	45.01 ± 16.35	45.97 ± 16.85	0.02
Gender (%)				
Male	49.51	/	/	
Female	50.49	/	/	
Race/Ethnicity (%)				0.70
Non-Hispanic White	62.19	64.27	64.11	
Non-Hispanic Black	10.98	10.60	11.36	
Mexican American	8.96	9.25	8.68	
Other race	15.87	15.89	15.85	
BMI	28.31 ± 7.23	28.24 ± 7.01	28.38 ± 7.45	0.45
Education (%)				0.01
less than high school	15.85	17.43	14.28	
high school graduate or equivalent	20.41	20.84	19.98	
college or AA degree	33.07	31.56	34.57	
college graduate or above	30.67	30.17	31.17	
Creatinine (mg/dL)	0.83 ± 0.25	0.83 ± 0.25	0.83 ± 0.25	0.95
Triglycerides (mg/dL)	140.55 ± 114.28	141.93 ± 119.70	139.21 ± 108.70	0.35
Cholesterol (mg/dL)	184.33 ± 42.24	184.37 ± 42.94	184.29 ± 41.54	0.94
Dietary energy (kcal)	2124.74 ± 1015.25	2132.87 ± 1007.45	2116.77 ± 1022.77	0.54
Dietary protein (g)	81.90 ± 45.71	81.94 ± 43.99	81.86 ± 47.33	0.95
Dietary sugars (g)	111.16 ± 75.50	112.30 ± 77.84	110.05 ± 73.11	0.25
Dietary fiber (g)	16.57 ± 10.69	16.90 ± 10.63	16.25 ± 10.73	0.02
Vitamin B6 (mg)	2.03 ± 1.87	1.99 ± 1.40	2.08 ± 2.24	0.06
Dietary folate (ug)	394.34 ± 260.11	399.61 ± 259.31	389.17 ± 260.78	0.12
Physical activity (MET)	1039.17 ± 4449.50	1140.86 ± 5525.55	939.48 ± 3042.04	0.08
Drinkers (%)	78.43	86.99	69.66	<0.01
Smoking habits (%)				0.02
Never	60.78	62.34	59.27	
Current	18.94	17.43	20.41	
Former	20.28	20.23	20.32	
Hypertension (%)	30.05	29.30	30.78	0.23
Diabetes (%)	10.06	9.51	10.60	0.15

**p Value** refer to the significance of the difference between males and females.

### Dietary vitamin C and SUA levels

**Fig 1:**
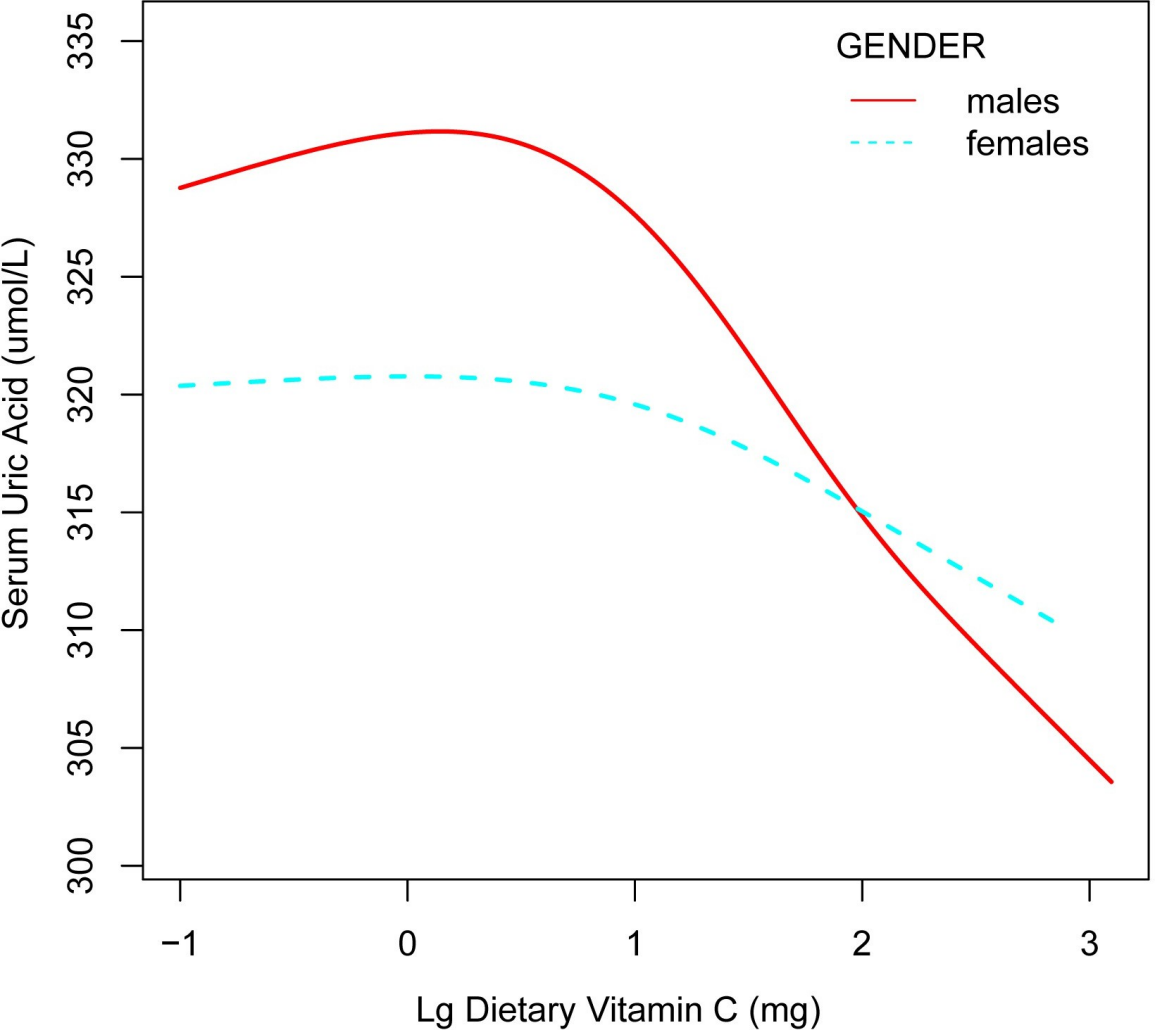
Association between dietary vitamin C (log transformed) and serum uric acid among US adults aged ≥20 years. The solid red line represents males, the blue punctate line represents females. Adjust for: age; race; BMI; education level; marital status; creatinine; cholesterol; triglycerides; hypertension; diabetes; dietary energy; dietary protein; dietary sugars; dietary fiber; Vitamin B6; dietary total folate; smoking habits; alcohol consumption; physical activity.

There was a negative relationship between dietary VC (log transformed) and SUA levels in model 0, model 2 (Table 2). According to the gender-specific subgroup analysis, there was a negative correlation between dietary VC and SUA levels in males. The P for interaction based on gender was 0.05, 0.13 and 0.02 in three models, respectively. From smooth curve fitting analysis (Fig 1), a negative linear relationship was found in males between dietary vitamin C (log transformed) and SUA.

**Table 2:** Associations between dietary vitamin C and serum uric acid levels among US adults aged ≥20 years.

	Total	Males	Females	P for interaction
Model 0	−4.42 (−7.90, −0.94)	−7.85 (−12.67, −3.03)	−0.85 (−5.88, 4.18)	0.05
Model 1	−2.72 (−6.07, 0.62)	−5.10 (−9.73, −0.47)	−0.01 (−4.85, 4.82)	0.13
Model 2	−7.27 (−11.58, −2.97)	−11.81 (−17.73, −5.89)	−2.33 (−8.62, 3.97)	0.02

Values are β-coefficients (95% CIs)

P for interaction refer to the significance of the difference between males and females

Model 0 adjusted for: None

Model 1 adjusted for: age; race; BMI.

Model 2 adjusted for: age; race; BMI; education level; marital status; creatinine; cholesterol; triglycerides; hypertension; diabetes; dietary energy; dietary protein; dietary sugars; dietary fiber; Vitamin B6; dietary total folate; smoking habits; alcohol consumption; physical activity.

### Dietary vitamin C and hyperuricemia risk

The relationship between dietary VC (log transformed) and hyperuricemia risk was negatively linked (Table 3). Stratified by gender, the P for interaction was 0.02, 0.04 and 0.04 in the three models, respectively, and the inverse correlations were more significant in males compared to females.

**Table 3:** Associations between dietary vitamin C intake and hyperuricemia risk among US adults aged ≥20 years.

	Total	Males	Females	P for interaction
Model 0	0.81 (0.73, 0.91)	0.68 (0.56, 0.82)	0.89 (0.78, 1.02)	0.02
Model 1	0.83 (0.74, 0.93)	0.71 (0.59, 0.87)	0.91 (0.79, 1.05)	0.04
Model 2	0.68 (0.57, 0.81)	0.56 (0.42, 0.74)	0.79 (0.63, 1.00)	0.04

Values are ORs (95% CIs)

P for interaction refer to the significance of the difference between males and females

Model 0 adjusted for: None

Model 1 adjusted for: age; race; BMI.

Model 2 adjusted for: age; race; BMI; education level; marital status; creatinine; cholesterol; triglycerides; hypertension; diabetes; dietary energy; dietary protein; dietary sugars; dietary fiber; Vitamin B6; dietary total folate; smoking habits; alcohol consumption; physical activity.

#### Supplemental vitamin C and SUA levels, hyperuricemia risk

**Fig 2:**
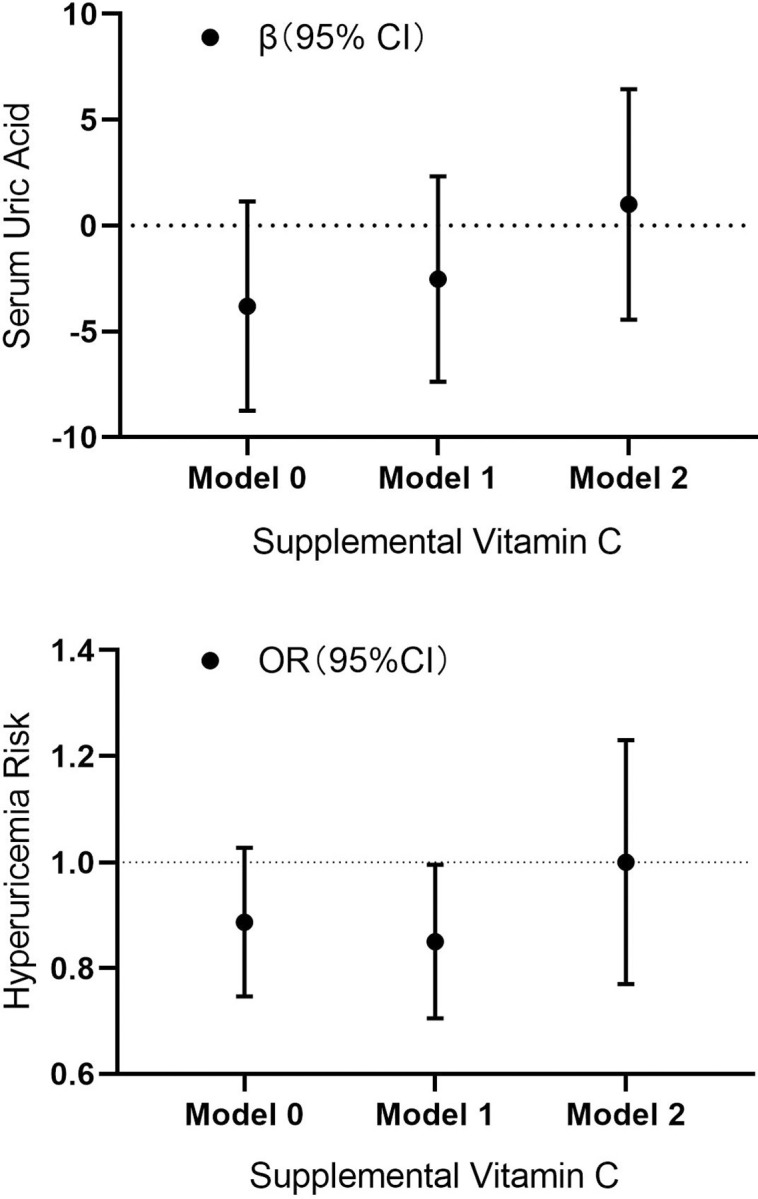
Associations of supplemental vitamin C with SUA levels and hyperuricemia risk. Model 0 adjusted for: none. Model 1 adjusted for: age, race, gender, and BMI. Model 2 adjusted for: age, race, gender, BMI, education level, marital status, creatinine, cholesterol, triglycerides, hypertension, diabetes, dietary energy, dietary protein, dietary sugars; dietary fiber, vitamin B6, dietary total folate, dietary vitamin C, smoking habits, alcohol consumption, and physical activity.

Among all participants, supplemental vitamin C was divided according to the categorical variables of "non-supplement" (supplemental VC = 0 mg) and "supplements" (supplemental VC > 0 mg). The baseline characteristics were presented in S1 Table. In the linear regression, compared with non-supplement, supplements group revealed no significance difference between supplemental VC and SUA levels. The results were −3.80 (95% CI: −8.75, 1.14), −2.52 (95% CI: −7.38, 2.33) and 1.00 (95% CI: −4.44, 6.44). In the logistic regression model, no significant differences were observed between supplemental VC and hyperuricemia risk. The results were 0.88 (95% CI: 0.75, 1.03), 0.84 (95% CI: 0.71, 1.00) and 0.98 (95% CI: 0.78, 1.24), as shown in Fig 2. Stratified by gender, no significant correlation was revealed.

Furthermore, the mean of VC supplements was 296 mg. The dose of supplemental vitamin C intake was separated by 300 mg, i.e., low-dosage supplements (0.1–300 mg), N = 966 and high-dosage supplements (>300 mg), N = 322. Compared with non-supplements, neither low-dosage supplements nor high-dosage supplements were associated with a reduced hyperuricemia risk (Table 4).

**Table 4:** Associations between dose of supplemental vitamin C and hyperuricemia risk.

	Non-supplement	Supplements (0.1-300mg)	Supplements (>300mg)
Model 0	ref	0.87 (0.73, 1.05)	0.89 (0.66, 1.20)
Model 1	ref	0.81 (0.67, 0.99)	0.94 (0.69, 1.29)
Model 2	ref	0.97 (0.75, 1.25)	1.04 (0.69, 1.56)

Values are ORs (95% CIs)

Model 0 adjusted for: None

Model 1 adjusted for: age; race; gender; BMI.

Model 2 adjusted for: age; race; gender; BMI; education level; marital status; creatinine; cholesterol; triglycerides; hypertension; diabetes; dietary energy; dietary protein; dietary sugars; dietary fiber; Vitamin B6; dietary total folate; dietary vitamin C; smoking habits; alcohol consumption; physical activity.

## Discussion

In this large sample study, we found significant negative associations between dietary VC and SUA levels in US adults. In the subgroup analysis stratified by gender, there was negative correlations between dietary VC and SUA levels in males. In addition, there were negative correlations between dietary VC and hyperuricemia risk, especially in males compared to females determined through an interaction test. However, there was no clear associations of supplemental VC with SUA levels and hyperuricemia risk. There was no relationship between high-dosage supplemental vitamin C (>300 mg) and hyperuricemia risk.

Numerous studies demonstrated that vitamin C intake was negatively correlated with SUA or hyperuricemia [21, 22, 30]. However, analysis of dietary VC and supplemental VC with SUA levels and hyperuricemia risk at the same study had hardly been published in the literature. In our study, the mean dietary VC intake was 81.66 ± 91.27 mg, 83.45 ± 94.96 mg for male and 79.90 ± 87.46 mg for female. In the United States and Canada, the current recommended dietary allowance for VC is 90 mg/day for adult men and 75 mg/day for adult women [31]. Dietary VC intake in our study was close to recommended value, and rational dose range were more meaningfully interpreted for quantiles of dietary VC. Because of the fact that dietary VC intake was hard to evaluate, the numbers of study between dietary VC and SUA levels were much less than supplemental VC related researches, and cross-sectional analysis was the main type of studies. In Korean study, dietary VC was not inversely related to SUA levels, but negatively with hyperuricemia risk [21]. This study did not investigate the relationship between supplemental VC and SUA levels, and focused on Korean population. The results of Korean study might not be suitable for Western populations. In genome-wide association studies (GWAS) of SUA level, the number of significant loci differed substantially. East Asian population had four loci (SLC2A9, ABCG2, SLC22A12 and MAF), African-American population had three loci (SLC2A9, SLC22A12 and SLC2A12), and European population had 28 loci (including SLC2A9, ABCG2, GCKR, et al) [32]. The distribution of loci revealed variation on different racial groups. Another report on NHANES showed dietary VC was inversely associated with hyperuricemia [30], but they did not investigate the relationship between dietary VC and SUA levels in normal. The negative relationships also existed in our study between dietary VC and hyperuricemia risk, regardless of whether male or female. However, interaction tests exhibited differences between groups stratified by gender, and more significant negative relationships in male.

There is no clear evidence that vitamin C prevents hyperuricemia [19]. In our study, there was no clear associations of supplemental VC with SUA levels and hyperuricemia risk, whether it is the comparison of the supplements group with the non-supplement group or the comparison of the high-dosage and low-dosage groups with the non-supplement group. A meta-analysis involving 1013 participants demonstrated that supplemental VC had impact on decreasing SUA levels [23]. Researchers also said placebo use, intervention duration, and study quality all affected the outcome. Previous intervention study found that supplemental VC did not influence SUA [24]. However, most of these studies did not perform plasma VC concentrations assessment. There was evidence that diet may not be the only factor determining systemic concentrations of VC based on genetic associations; variations in genes involved in redox homeostasis and VC transport were related with plasma levels [31]. Plasma VC levels would reach a plateau at about 200 mg/d VC intake, plasma VC levels would not ascend even upon interventions with higher doses of VC intake [31, 33]. Briefly, a joint of gene and diet decides the plasma VC levels, supplemental VC might not show negative association with SUA in high plasma VC levels. Gender and genetic differences are unchangeable, and the benefits of nutritional supplementation are still controversial. Adequate dietary vitamin C could be our best choice.

As known for antioxidative properties, VC and SUA help maintain balance in humans from a molecular mechanism point of view [34]. SUA acts as antioxidant in deficiency of plasma VC. The correlation between high plasma VC and low SUA was affirmed. Due to genetic variations in SLC23A1, plasma VC levels could rise or fall in current studies [32]. However, the SLC23A1 genetic variant causing long-term high plasma VC was not decrease SUA levels or hyperuricemia risk in Mendelian randomization study including 106,147 individuals [35]. There is about 60% uric acid involved in metabolism, and the rest 40% is excreted by the gut and urethra. Excretion through the urethra is the primary method. A series of urate transporters including SLC (GLUT9, URAT1, OAT1, OAT2) and ABC (ABCG2) transporters expressed in urethra, especially the proximal convoluted tubules, maintain urate homeostasis [36]. But it is not clear how vitamin C lowers SUA. This feature of VC may be explained by reabsorption transport of uric acid in renal tubules [37] and the glomerular filtration rate increasing [38]. The uric acid-lowering effect of vitamin C is related to activity of URAT1 and two sodium-dependent anion cotransporters SLC5A8/SMCT1 and SLC5A12/SMTC2 located in the proximal tubular epithelial cells [19, 39]. The mechanisms remain elusive and require further study.

The major strengths of this study should be highlighted as follows. First, based on the NHANES database, a large population (6308 subjects) was included, with nationally representative samples among US adults. All data generated from the NHANES database were obtained by standard procedures and trained staff, which improved the accuracy and effectiveness of the study. Second, strict exclusion criteria were used in our study; medications including hydrochlorothiazide and allopurinol that affect SUA concentrations were excluded. Third, thanks to adequate samples, we performed subgroup analyses, illustrating that effects of dietary VC intake on hyperuricemia were different from gender specific. There were limitations of this study. First, because it is a cross-sectional study, we cannot assess whether SUA changed with vitamin C intake over time, nor can we determine the causality. Second, we adjusted many variables. However, other missing confounding variables might result in bias as well. Third, there is no single ideal strategy for evaluating dietary intake data in surveys. Thus, dietary data collection may be prone to measurement bias. Fourth, in our study, the proportion of hyperuricemia in each gender differs from the previous. This might be related to our exclusion criteria. But, the results of the association between dietary vitamin C (log transformation) and hyperuricemia risk were similar (shown in S2 Table), if we defined hyperuricemia as SUA concentrations >420 umol/L in males and females.

## Conclusions

This study indicated the negative associations of dietary vitamin C intake with SUA levels and hyperuricemia risk among US adults. The inverse correlations between dietary vitamin C and SUA levels and hyperuricemia risk were more significant in males compared to females. There were no associations between supplemental vitamin C and SUA levels or hyperuricemia risk. The relationship should be further investigated with a prospective population-based cohort study.

## Supporting information

S1 Checklist STROBE statement—checklist of items that should be included in reports of observational studies. (DOCX)

S1 Table Characteristics of the participants according to supplemental vitamin C. (DOCX)

S2 Table Associations between dietary vitamin C intake and hyperuricemia risk among US adults aged ≥20 years. (DOCX)
