# Supplementary material for: Associations between vitamin C intake and serum uric acid in US adults: Findings from National Health and Nutrition Examination Survey 2011–2016
Source: PLoS One. 2023 Oct 13;18(10):e0287352. doi: 10.1371/journal.pone.0287352 (PMC10575504; doi:10.1371/journal.pone.0287352)
Supplement: S1 Table — (DOCX) [file pone.0287352.s002.docx]

**Table S1**. Characteristics of the participants according to supplemental vitamin C.

|  | Non-supplement (N=5020) | Supplements (N=1288) | P value |
| --- | --- | --- | --- |
| Supplemental Vitamin C (mg) | 0.00 ± 0.00 | 296.28 ± 506.32 | <0.01 |
| Dietary vitamin C (mg) | 80.36 ± 90.93 | 85.79 ± 92.21 | 0.05 |
| SUA (umol/L) | 316.27 ± 81.55 | 312.68 ± 77.51 | 0.14 |
| Hyperuricemia （%） | 18.47 | 17.45 | 0.895 |
| Age (years) | 43.74 ± 16.09 | 51.08 ± 17.01 | <0.01 |
| BMI | 28.36 ± 7.20 | 28.14 ± 7.33 | 0.31 |
| Male （%） | 50.98 | 44.81 | <0.01 |
| Race/ethnicity (%) |  |  | <0.01 |
| Non-Hispanic White | 60.76 | 75.16 |  |
| Non-Hispanic Black | 12.18 | 7.15 |  |
| Mexican American | 10.22 | 4.94 |  |
| Other Race | 16.84 | 12.75 |  |
