# Supplementary material for: Associations between vitamin C intake and serum uric acid in US adults: Findings from National Health and Nutrition Examination Survey 2011–2016
Source: PLoS One. 2023 Oct 13;18(10):e0287352. doi: 10.1371/journal.pone.0287352 (PMC10575504; doi:10.1371/journal.pone.0287352)
Supplement: S2 Table — (DOCX) [file pone.0287352.s003.docx]

**Table S2**. Associations between dietary vitamin C intake and hyperuricemia risk among US adults aged ≥20 years.

|  | **Total** | **Males** | **Females** | **P for interaction** |
| --- | --- | --- | --- | --- |
| Model 0 | 0.89 (0.76, 1.04) | 0.72 (0.58, 0.89) | 1.10 (0.88, 1.38) | ＜0.01 |
| Model 1 | 0.92 (0.78, 1.08) | 0.74 (0.59, 0.93) | 1.15 (0.91, 1.46) | ＜0.01 |
| Model 2 | 0.72 (0.57, 0.92) | 0.57 (0.41, 0.79) | 0.94 (0.66, 1.35) | ＜0.01 |

P for interaction refer to the significance of the difference between males and females

Model 0 adjusted for: None

Model 1 adjusted for: age; race; BMI.

Model 2 adjusted for: age; race; BMI; education level; marital status; creatinine; cholesterol; triglycerides; hypertension; diabetes; dietary energy; dietary protein; dietary sugars; dietary fiber; Vitamin B6; dietary total folate; smoking habits; alcohol consumption; physical activity.
